# Utilizing mouse optic nerve crush to examine CNS remyelination

**DOI:** 10.1016/j.xpro.2021.100796

**Published:** 2021-09-09

**Authors:** Tracey A.C.S. Suter, Jing Wang, Huyan Meng, Zhigang He

**Affiliations:** 1 Boston Children’s Hospital | Harvard Medical School, Boston, MA 02115, USA

**Keywords:** Model Organisms, Molecular biology, Gene expression, Antibody, Neuroscience

## Abstract

In developing pro-myelination treatment, an important hurdle is the lack of reliable animal models for assessing de novo myelination in disease settings. We recently showed that regenerated axons in injured optic nerves fail to be myelinated, providing an animal model for this purpose. Here, we describe procedures to promote axonal regeneration, administer optic nerve crush, and assess oligodendrocyte differentiation and maturation into myelination-competent oligodendrocytes. This protocol allows for testing the efficacy of remyelination treatments in an *in vivo* central nervous system (CNS).

For complete details on the use and execution of this protocol, please refer to Wang et al. (2020) and Bei et al. (2016).

## Highlights

• Optic nerve crush provides a clear baseline model, where little to no spontaneous remyelination occurs on regenerated axons without a remyelination treatment.
• Method is highly reproducible and provides a consistent time course and injury region for pathology analysis.
• These methods are valuable for testing treatments or manipulations of altering remyelination.
• These protocols permit the examination and quantification of the proportion of OPCs and newly generated oligodendrocytes post injury and the level of myelination.

## Graphical abstract

**Figure:**
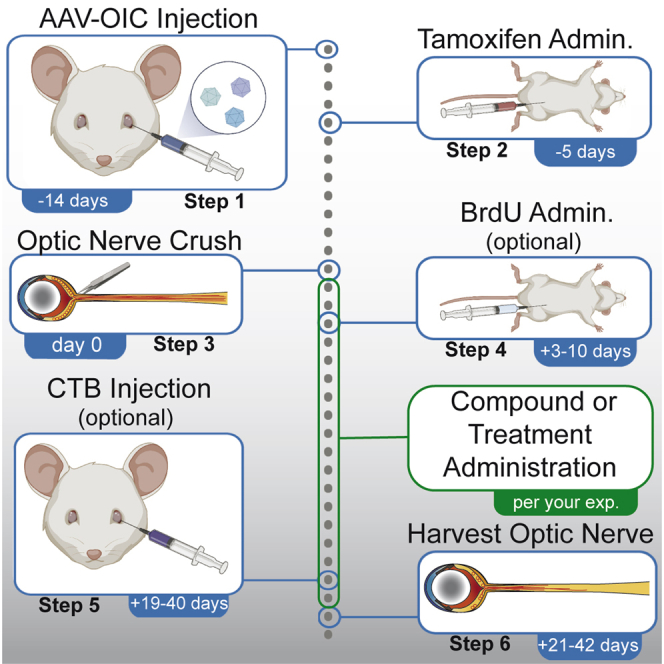

## Teaser

In developing pro-myelination treatment, an important hurdle is the lack of reliable animal models for assessing de novo myelination in disease settings. We recently showed that regenerated axons in injured optic nerves fail to be myelinated, providing an animal model for this purpose. Here, we describe procedures to promote axonal regeneration, administer optic nerve crush, and assess oligodendrocyte differentiation and maturation into myelination-competent oligodendrocytes. This protocol allows for testing the efficacy of remyelination treatments in an *in vivo* CNS system.

## Before you begin

**Figure 1:**
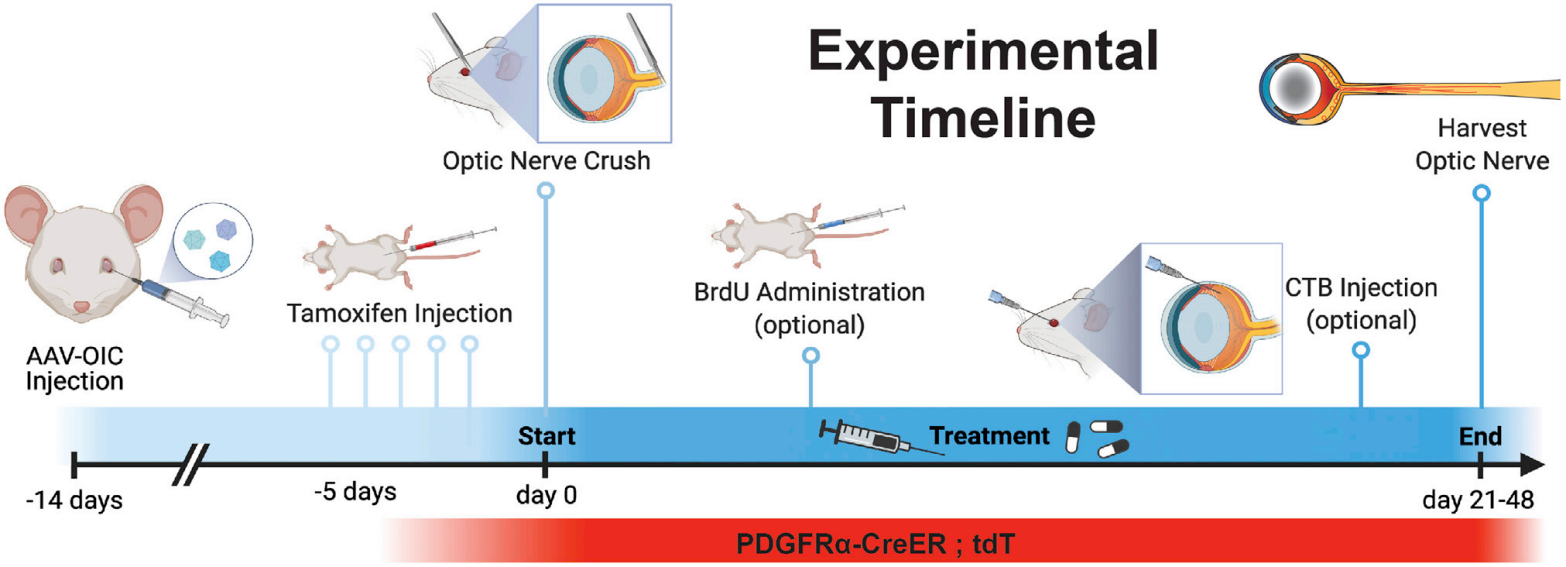
Timeline of the protocol Schematic showing the major steps of this protocol in chronological order and their purpose in the overall protocol.

Please familiarize yourself with the workflow pipeline illustrated in Figure 1 and the graphical abstract before you begin. There are many steps to this protocol but not all are required. Determine what you wish your final readout to be before starting.

### Part 0: Breeding of PDGFRα-CreER;tdTomato mice.

**Timing: [weeks]**

1. Cross heterozygous PDGFRα^CreER/+^ mice (Kang et al., 2010) with homozygous tdTomato (Arenkiel et al., 2011) mice to generate PDGFRα^CreER/+^;tdTomato^+/-^ mice for any lineage tracing experiments.
2. Use male or female mice that are between 6–8 weeks of age at time of optic nerve crush.

***Note:*** Do not use homozygous (PDGFRα^CreER/CreER^) animals for breeding or experimental purposes. Please refer to JAX lab breeding information of this mice strain. See troubleshooting 1**.**

### Part 1: Intravitreal AAV injection for axon regeneration

**Timing: [0.5 days]**

**Figure 2:**
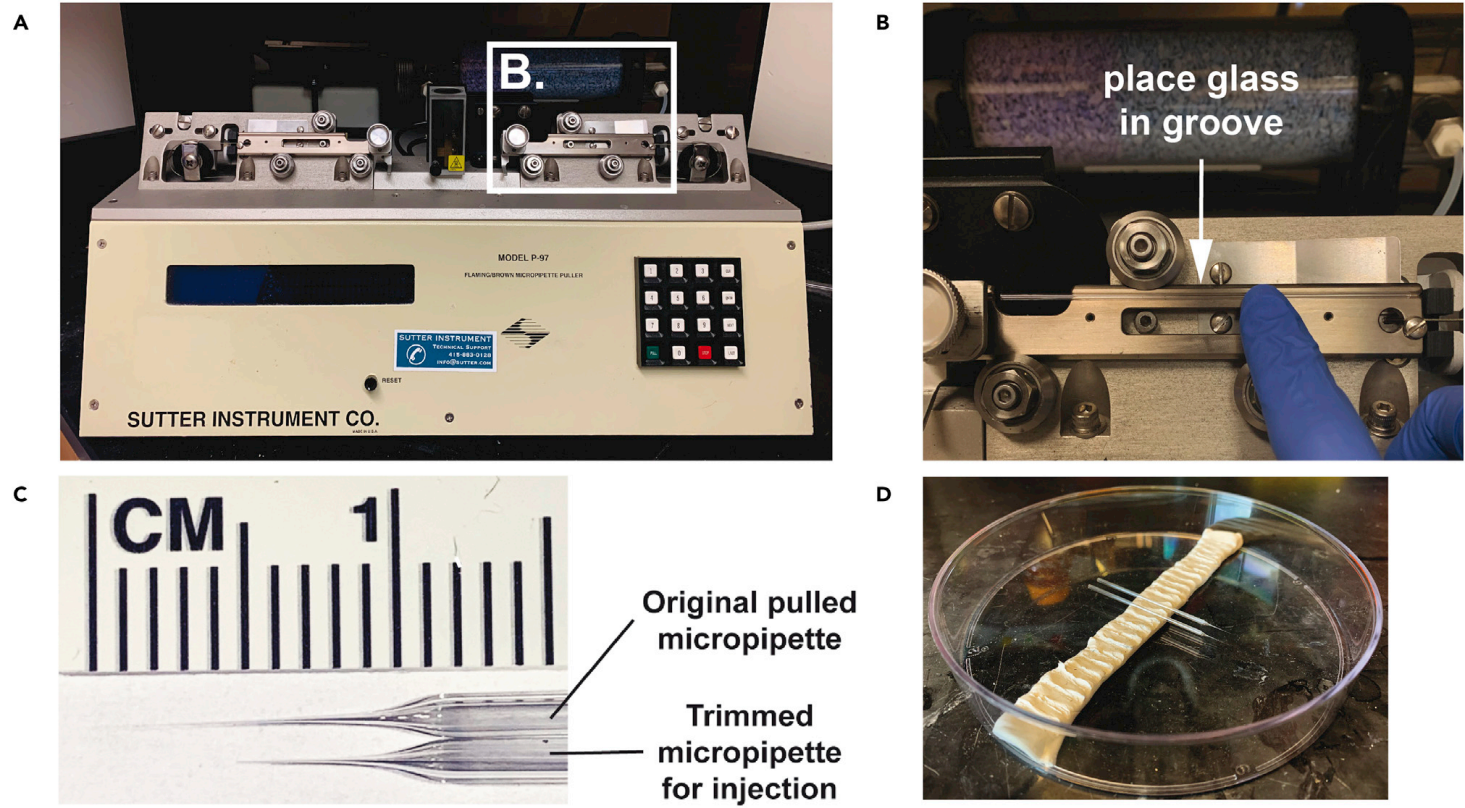
Glass micropipette preparation (A) Sutter Instruments Co. Pipette puller, White box indicates region shown in B. (B) Highlights the location of the groove where the Borosilicate Glass gets placed to prevent accidentally touching the filament. (C) The top glass micropipette shows the structure of needle upon initially pulling under conditions described in Step 4 (Part 1 in before you begin). The bottom micropipette shows the approximate length and structure of the needle after trimming. The lower needle should be used for intravitreal injections. (D) Shows the proper storage of the microinjection needles.

3. Prepare anesthetic (See materials and equipment)
  a. Final desired concentration of Ketamine (100–120 mg/kg)
  b. Final desired concentration of Xylazine (10 mg/kg)
  c. Mix Ketamine and Xylazine with saline to get final concentration.

4. Generate pulled glass micropipettes (Figure 2):
  a. Need Micropipette puller to make microinjection needles
    i. We use a Sutter Flaming/Brown Micropipette Puller (P-97)
    ii. Get fire-polished Sutter Borosilicate Glass: Outer Diameter: 1.5 mm; Inner Diameter: 0.86 mm; Length 10 cm
  b. Turn on and open machine and set up pull parameters:
    i. Settings: ‘P = 500; ‘Heat’ = 528; ‘Pull’ = 20; ‘Velocity’ = 35; ‘Time’ = 100 (1/2 ms)
    ii. Settings should generate a pipette tip with an approximate resistance of 50 MΩ (prior to cutting the needle – see Step 4j below).
  c. Place a Borosilicate Glass into groove of the 1^st^ micropipette puller (Figure 2B), and then slide glass towards the environmental chamber (which surrounds heating filament) until end of glass enters and passes through chamber to the other side (until start of glass fills 4/5^th^ of space)
  d. Tighten the screw of the 1^st^ micropipette puller to secure the Borosilicate glass in place
  e. Release puller (with glass held in place) brake, and slide 1^st^ puller towards environmental chamber until glass enters 2^nd^ puller on other side, and until 1^st^ puller meets chamber
  f. Hold 1^st^ puller in place, release 2^nd^ puller, and move 2^nd^ puller until it reaches chamber
  g. Tighten screw of 2^nd^ puller to hold Borosilicate glass in place between both pullers
    ***Note:*** Ensure glass fills equal amount of space (∼4/5^th^) in gap of each micropipette puller
  h. To generate two symmetrical microinjection needles, close lid of machine and press ‘pull’.
  i. Holding needle, loosen screw of puller, and then carefully remove each needle
  j. Inspect each needle tip, and then cut tip (at a 45º angle) to a length of ∼6 mm (Figure 2C).
  k. Store microinjection needles in 15 cm plate with tape or sticky tack to keep needles securely in place and preserve needle integrity (Figure 2D).
    ***Note:*** Aseptic technique requires that only the tip of needle needs to remain sterile

5. Ensure you have all the necessary reagents, including the three AAVs needed to induce axonal regeneration:
  a. Obtain the following three AAVs (AAV-OIC), which will promote retinal ganglion cell (RGC) axonal regeneration(Beiet al., 2016; Wanget al., 2020):
    i. AAV2/2-CAG-OPN
    ii. AAV2/2-CAG-IGF
    iii. AAV2/2-CAG-CNTF
  b. Combine AAVs to single working dilution (1:1:1 mix)
  c. Ensure you have sufficient virus for all of your planned animals (∼1–2 μL per animal).
  d. Use minimum titer of 1.0 X 10^13^ GC/mL
  e. Keep virus on ice during the procedure
6. Fill Hamilton syringe and tubing with baby oil – be sure to avoid bubbles.

***Note:*** Baby oil provides a more resistance within the syringe than air or water, which allows for increased control over injection speed.

**CRITICAL:** Acquire approval from your institution’s ethical committee for vitreous injections procedures prior to starting this protocol.

### Part 2: Oligodendrocyte lineage reporter expression

**Timing: [0.5 days]**

7. Prepare Tamoxifen for 5 days of injections:
  a. Pre-warm 4.5 mL of sunflower oil at 42°C for 30 min in a sterile scintillation vial.
  b. While sunflower oil warms, allow the tamoxifen to reach room temperature.
  c. Mix 500 μL of ethanol into the pre-warmed sunflower oil.
  d. Add 100 mg of tamoxifen to the warmed sunflower oil - ethanol (final tamoxifen concentration = 20 mg/mL).
    ***Note:*** Tamoxifen is light sensitive, so be sure to make and store in a light-blocking vessel (either amber or foil wrapped).
  e. Place vial on rocker at 37°C for 1–2 h.
  f. Occasionally vortex solution (∼ every 15 min).
  g. After tamoxifen is in solution, store at 4°C for 3–4 days.

### Part 3: Optic nerve crush (ONC)

**Timing: [1 h]**

8. Prepare anesthetic (See before you begin for Part 1, step 3, above).
9. Obtain post-operative care materials per your institute’s ethics protocol.
10. Sterilize all surgical materials.

**CRITICAL:** Acquire approval from your institution’s ethical committee for optic nerve crush procedures prior to starting this protocol.

***Optional:*** BrdU solution preparation may not be necessary for your experiment. If not, please omit Part 4 and proceed to Part 5.

### Part 4: BrdU solution preparation

**Timing: [0.5 h]**

11. (Optional) Prepare BrdU for injection.
  a. Final concentration of BrdU = 100 mg/kg per animal
  b. Add BrdU to sterile 1**×**PBS and place in 37°C water bath
  c. Vortex occasionally until BrdU is in solution

***Note:*** BrdU solution can be aliquoted and stored at 4°C for 5 days and at −20°C for 3 months. Avoid repeated freeze-thaws. You may also use EdU in place of BrdU.

***Optional:*** Regenerated axon labeling may not be necessary for your experiment. If not, please omit Part 5 and proceed to Part 6.

### Part 5: Label regenerated axons

**Timing: [30 min]**

12. Make AlexaFluor Conjugated Cholera Toxin subunit β (CTB) solution (1 μg/μL) (See materials and equipment).
  a. Add 500 μL of sterile saline to 500 μg of CTB.
  b. Prepare 100 μL aliquots and store at −20°C

***Note:*** Be sure to be aware of the reporter line used to label the Oligodendrocyte Progenitor Cells (OPC) linage. Choose a fluorophore that is not the same as the one used for the endogenous reporter.

13. Prepare anesthetic (See ‘before you begin’ from Part 1, step 3 above).
14. Prepare Hamilton Syringe and tubing with baby oil (See ‘before you begin’ Part 1, step 6 above).
15. Generate micropipettes (See ‘before you begin’ Part 1, step 4 above and Figure 2)

### Part 6: Optic Nerve Dissection

**Timing: [1 h]**

16. Prepare anesthetic (See ‘before you begin’ Part 1, step 3 above).
17. Make 4% Paraformaldehyde (PFA) in 1×PBS for perfusion
  a. Store at 4°C.
  b. Make sure PFA solution is no more than 1 week old.

### Part 7: Immunohistochemistry & analysis

**Timing: [10–15 min]**

18. Make Blocking buffer solution:
  a. 0.5% Triton X-100 and 10% normal donkey serum in 1×PBS
19. Ensure access to confocal microscopy and Image J or other imaging analysis software.

## Key resources table

**Table:** 

REAGENT or RESOURCE	SOURCE	IDENTIFIER
**Antibodies**		

Rabbit anti-Olig1 (1:50)	Dr. Charles D Stiles	N/A
Rabbit anti-Olig2 (1:300)	Novus biologicals	NBP1-28667
Rat anti-PDGFRα (CD140a) (1:100)	BD Bioscience	558774
Mouse anti-CC1 (APC) (1:100)	Millipore	OP80
Mouse anti-GSTπ (1:100)	BD Transduction Laboratories	610718
Rat anti-BrdU (1:300)	Abcam	ab6326
Rabbit anti-P2Y12 (1:500)	AnaSpec	AS-55043A
Rabbit anti-ASPA (1:500)	Millipore	ABN1698
Mouse anti-Olig2 (Conjugated) (1:250)	Millipore	MABN50A4
Rat anti-MBP (1:300)	Abcam	ab7349
Mouse anti-MAG (1:100)	Millipore	MAB1567
Rabbit anti-RFP (1:500)	Abcam	Ab34771
Chicken anti-GFP (1:200)	Axes Lans, Inc	GFP-1020
Mouse anti-Ankyrin-G (1:50)	Antibodies Incorporated	75-146
Rabbit anti-Caspr (1:1000)	Abcam	ab34151

**Bacterial and virus strains**		

pAAV-CAG-Cre	BCH Viral Core	N/A
pAAV-CAG-IGF1	BCH Viral Core	N/A
pAAV-CAG-CNTF	BCH Viral Core	N/A
pAAV-CAG-OPN	BCH Viral Core	N/A

**Chemicals, peptides, and recombinant proteins**		

Ketamine	Controlled Substance – contact your animal facility veterinarian	N/A
Xylazine	Controlled Substance – contact your animal facility veterinarian	N/A
Buprenorphine	Controlled Substance – contact your animal facility veterinarian	N/A
1× PBS	Boston BioProducts	BM-220
Triton X-100	Sigma	T8787-50ML
Normal donkey serum	Jackson ImmunoResearch	017-000-121
Alexa-conjugated cholera toxin subunit B	Thermo Fisher Scientific	C34776
Fluoromount-G with DAPI	SouthernBiotech	0100-20
Tamoxifen	VWR	IC15673883
Bromodeoxyuridine (BrdU)	Sigma	B5002-1G
5-ethynyl-2′-deoxyuridine (EdU)	Thermo Fisher Scientific	A10044
sterile saline	Boston BioProducts	BM-220
Sunflower oil	Sigma	8001-21-6
5% Dextrose & 0.9% NaCl	DC Dental Express	SKU: 804-L6101

**Critical commercial assays**		

HCR v3.0 kits	Molecular Instruments	
Click-iT™ EdU Cell Proliferation Kit for Imaging, Alexa Fluor™ 647 dye	Thermofisher	C10340

**Experimental models: Organisms/strains**		

Mouse: C57BL/6	The Jackson Laboratory	Stock No: 000664
Mouse: PDGFRα-CreER	The Jackson Laboratory	Stock No: 018280
Mouse: Rosa26-STOP-tdTomato mice	Fan Wang Lab	(Arenkiel et al., 2011)
Mouse: PLP-CreER	The Jackson Laboratory	Stock No: 005975
Mouse: Tau-GFP	The Jackson Laboratory	Stock No: 021162

**Oligonucleotides**		

PDGFRα-CreER primer 1	TCA GCC TTA AGC TGG GAC AT	Transgene Forward
PDGFRα-CreER primer 2	ATG TTT AGC TGG CCC AAA TG	Transgene Reverse
PDGFRα-CreER primer 3	CTA GGC CAC AGA ATT GAA AGA TCT	Internal Positive Control Reverse
PDGFRα-CreER primer 4	GTA GGT GGA AAT TCT AGC ATC ATC C	Internal Positive Control Reverse
RTM – Rosa/01 primer 1	CACTTGCTCTCC CAAAGTCG	(Arenkiel et al., 2011)
RTM - Rosa/02 primer 2	TAGTCTAACTCGCGACACTG	With Rosa/01 detects WT band (~560bp)
RTM – CAG/02 primer 3	GTTATGTAACGCGGAACTCC	With Rosa/01 detects RTM allele (~300bp)
PLP-CreER primer 1	AGG TGG ACC TGA TCA TGG AG	Transgene Forward (~440 bp)
PLP-CreER primer 2	ATA CCG GAG ATC ATG CAA GC	Transgene Reverse
PLP-CreER primer 3	CTA GGC CAC AGA ATT GAA AGA TCT	Internal positive control Forward (324 bp)
PLP-CreER primer 4	GTA GGT GGA AAT TCT AGC ATC ATC C	Internal positive control Reverse.

**Software and Algorithms**		

ImageJ	NIH	RRID: SCR_003070
Prism – GraphPad	GraphPad	Prism 8

**Other**		

Confocal microscope	Zeiss	LSM 710
Sutter Flaming/Brown Micropipette Puller	Sutter Instruments	P-97
Sutter Borosilicate Glass	Sutter Instruments	B150-86-10
Bulldog Serrefines	F.S.T.	18051-28
Hamilton Syringe	Hamilton	80975
Plastic Tubing	McMaster-Carr	1883T1 & 1883T4
Student Vanna spring scissors	F.S.T.	91501-09
Superfrost Plus Gold	Fisher Brand	15-188-48

## Materials and equipment

**Table:** **Anesthetic:** Ketamine and Xylazine

Reagent	Final concentration	Amount	Storage temperature
Ketamine	15% v/v	1 mL	Room temperature
Xylazine	5% v/v	0.5 mL	Room temperature
Saline		8.5 mL	Room temperature
**Total**		**10 mL**	Room temperature

**Storage:** Non-diluted Ketamine is stable for 1 year. Non-diluted Xylazine is stable for 2 years. Once combined and diluted, the shelf life is 6 months from the mix date.

**Final Concentration:** Ketamine 100 mg/mL, Xylazine 10 mg/mL

**CRITICAL:** These are controlled substances that should be obtained through your animal facility veterinarian. Please follow the instructions provided by your institution’s ethical committee.

**Table:** **Analgesic:** Buprenorphine (administered at 0.1 mg/kg)

Reagent	Final concentration	Amount
Buprenorphine (0.3 mg/mL)	0.03 mg/mL	1 Ml
5% Dextrose in Saline		9 Ml
**Total**		**10 Ml**

**Storage:** Diluted buprenorphine has a shelf life of two months at room temperature from when it is mixed.

**CRITICAL:** This is a controlled substance that should be obtained through your animal facility veterinarian. Please follow the instructions provided by your institution’s ethical committee.

**Table:** **Axonal Labeling:** Cholera Toxin Subunit β

Reagent	Final concentration	Amount
Cholera Toxin Subunit β	20 mg/mL	100 mg
Sterile Saline		5 mL
**Total**		**5 mL**

**Storage:** after dilated with saline, store at −20°C for 3 months.

**CRITICAL:** Protect from light whenever possible.

***Alternatives:*** ThermoFisher offers Cholera Toxin Subunit β conjugated to multiple fluorophores, just be sure to select one that will not interfere with the tdTomato expression.

**Table:** **CreER Induction:** Tamoxifen (administered at 100 mg/kg)

Reagent	Final concentration	Amount
Tamoxifen	20 mg/mL	100 mg
Sunflower Oil		4.5 mL
Ethanol	10% v/v	0.5 mL
**Total**		**5 mL**

**Storage:** the working solution can be aliquoted and stored at −20°C for 1 month. Avoid repeat freeze thaw.

**CRITICAL:** Tamoxifen may be irritating to the mucous membranes and upper respiratory tract.

May be harmful by inhalation or skin absorption. May cause cancer. May cause eye, skin, or respiratory system irritation.

**Table:** **Birth Dating Labeling**: BrdU - (100 mg/kg, intraperitoneal injection)

Reagent	Final concentration	Amount
BrdU	10mg/mL	100 mg
Sterile 1×PBS		10 mL
**Total**		**10 mL**

**Storage:** the working solution can be aliquoted and stored at −20°C for 1 month. Avoid repeat freeze thaw.

**CRITICAL:** BrdU can incorporate into DNA and can cause genetic defects. Be careful to not to inhale or have accidental exposure. Additionally, BrdU may be irritating to the mucous membranes and upper respiratory tract.

***Alternatives:* 1)** You may purchase pre-made BrdU that is already in solution and ready for use (ThermoFisher, Catalog # 000103). **2)** You may also use EdU in place of BrdU to label actively dividing cells. For processing of slides with EdU, we recommend the ThermoFisher Click-iT kit (ThermoFisher: C10340).

**Table:** **Blocking Buffer Solution:** 10% Donkey Serum and 0.5% Triton in PBS

Reagent	Final concentration	Amount
Donkey Serum	10%	10 mL
Triton X-100	0.5%	0.5 mL
1×PBS		89.5 mL
**Total**		**100 mL**

**Storage:** Keep at 4°C for 2 weeks.

## Step-by-step method details

### Part 1: Intravitreal AAV injection

**Timing: [0.5 day, ∼1 minute per eye for injection]**

**Figure 3:**
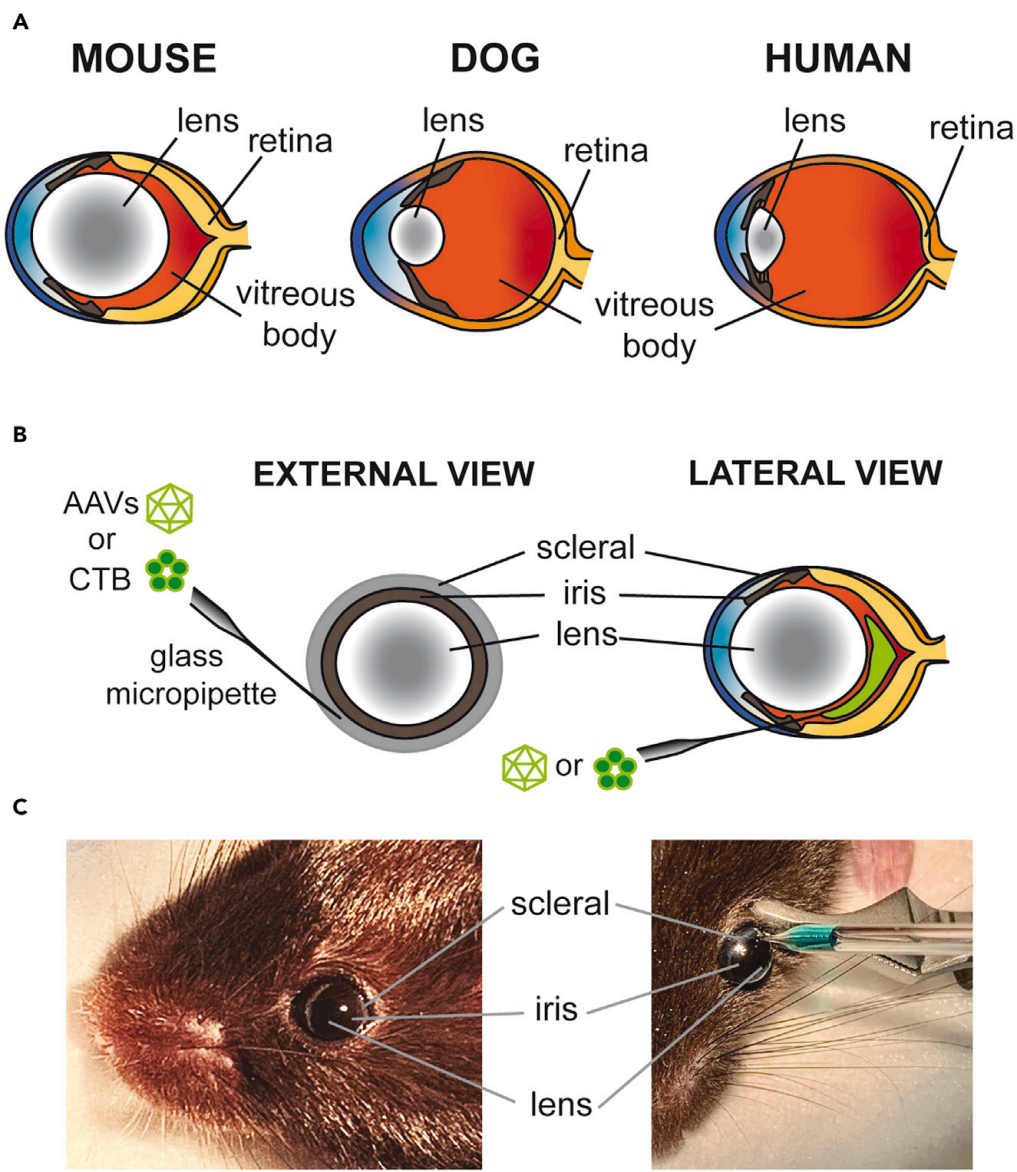
Anatomy of mouse eye and intravitreal injection site (A) Highlights the structural differences between the mouse eye and that of a dog or human, with the lens being significantly larger. (B) Indicates the site where the glass micropipette should be inserted into the eye for the injection of the AAVs to promote axonal regeneration or Cholera Toxin Subunit β (CTB). (C) Example images of the regions shown in the schematic in (B).
7. Place mouse head under dissection scope at a 60º angle and stabilize the head with non-dominant hand.
8. Using your dominant hand, insert needle at a parallel angle to the eye within the sclera (Figure 3) about 0.5 mm posterior to the limbus and into the vitreous body.
  a. For left eye, insert needle close to ear/clamp. For right eye, insert needle from nose side
  b. Ensure tip is angled towards back of eye, and slightly rotate tip during insertion to ease entry.

***Note:*** The lens of the mouse eye is very large, make sure the needle is at the periphery of the eye within the sclera, to avoid damaging the lens (see Figure 3)

1. Anesthetize the mouse with Ketamine and Xylazine
  a. Weigh the mouse (plan to administer 0.1 mL per gram)
    ***Note:*** Depending on your own experimental design, and whether you are interested in development or aging etc, mouse can be almost any age. This protocol, as written, describes how to conduct these procedures in mice > 4 weeks of age. We have not attempted this protocol in pups less than 4 weeks old.
  b. Administer proper dose of anesthetic via intraperitoneal (i.p.) injection
  c. Check for complete anesthetic state by tail or foot pinch for movement, or by the eye blink reflex.
    ***Note:*** A second injection at 1/3 to 1/2 of the original dose can be used is the animal is not under after 15 min. Follow your institute’s ethics protocol.

2. Attach microinjection needle (ensure the tip is sharp and narrow) to the prepared Hamilton syringe and tubing (See above).
3. Apply 1 drop of Tropicamide to each mouse eye to dilate pupil.
4. Apply 1 drop of Proparacaine to each mouse eye for local anesthesia.
5. Hold mouse head in left hand, so that its skin spreads away from eye.
6. Using a Bulldog Serrefines (FST, #18051-28) to clamp skin surrounding back of eye so that eye protrudes out for injections (Figure 3C).
  a. Open clip as wide as possible and approach the back (caudal) side of the eye.
  b. Line up tip of clip with outer edges of eye (at the widest point/diameter)
  c. Allow clip to close so the eye lid skin around the eye is pinched causing the eye to protrude out.

9. Remove ∼1–2 μL of vitreous humor from eye using Hamilton syringe and the micropipette.
  a. Aseptic technique requires using one needle to remove liquid and another for injection – do NOT attempt to ‘wash’ and reuse the needle, use a fresh one each time.

***Note:*** This is done to reduce spilling and intraocular pressure from the upcoming injection.

10. Fill new micropipette with 2–2.5 μL of pre-mixed AAVs using Hamilton syringe.
  a. Be careful to not get bubbles in the needle.
11. Inject AAVs into the eye
  a. Re-insert needle into back of eye (via hole from 1^st^ insertion)
  b. Slowly inject the 2 μL of AAVs into the intravitreal space
  c. Leave needle in place for a few seconds after injection to allow it to disperse in the eye and reduce AAV loss to spilling.
  d. Use cotton bud to remove any blood.
12. Rotate the mouse and repeat steps 6–11 on the 2^nd^ eye (if doing a bilateral ONC).
13. Apply antibiotic ointment (i.e., Vetropolucine) directly onto the mouse eyes from the tube.
  a. Be sure to cover the eye completely.
14. Monitor mice approximately every 15 min until fully recovered from anesthesia
  a. Check respiratory rate, hydration (by evaluating skin turgor), pain, and position of mice.
  b. Turn mouse from side to side every 30 min to prevent pulmonary congestion.
15. Remain with the animals until all mice awake and can then return cages to housing unit.
16. Monitor mice daily for 7–10 days and then twice a week for long term.

### Part 2: Oligodendrocyte lineage reporter expression

**Timing: [1 h]**

17. Begin to administer Tamoxifen 5 days prior to Optic Nerve Crush (below).
  a. Ensure tamoxifen powder is still in solution.
    ***Note:*** If tamoxifen has precipitated, warm in water bath and vortex until back into solution prior to administration.
  b. Can be administered via oral gavage or via i.p. injection
  c. Determine Injection dose by weight for each animal
    i. Use approximately 100 mg tamoxifen/kg body weight
    ii. For adult mice, a standard dose of 100 μL tamoxifen in sunflower oil solution is effective for inducing recombination.
    iii. If administering via i.p. sanitize injection site with 70% ethanol prior to injection,

18. Administer Tamoxifen once every 24 h for 5 consecutive days.

***Note:*** Throughout the course of tamoxifen injections and any post-injection wait period, mice should be closely monitored for any adverse reactions to the treatment. Also keep Tamoxifen solution protected from light whenever possible.

### Part 3: Optic nerve crush

**Timing: [0.5–1 day depending on the number of animals]**

***Note:*** Video of the optic nerve crush procedure can be found on *JOVE* (Tang *et al.*, 2011). Slight differences between their protocol and ours exist, but the overall procedural process is grossly similar.

19. Anesthetize the mouse with i.p. injection of ketamine and xylazine (See materials and equipment and AAV Intravitreal Injections above).
20. Apply eye ointment containing atropine sulfate to protect the cornea during surgery and administer first dose of 0.1 mg/kg Buprenorphine.

***Optional:*** Retract the eyelids slightly using surgical tape

21. Make a small incision through the dorsal conjunctival membranes at the 4 o’clock position around the eye
22. Expose the optic nerve intra-orbitally

***Note:*** The optic nerve will appear starkly white compared to the surrounding tissues within the eye socket.

23. Using jeweler’s forceps (Dumont #5, #11252-00) crush the optic nerve for 5 s approximately 1 mm behind the optic disc.

***Note:*** Do not to damage the underlying ophthalmic artery to ensure the preservation of the retinal blood supply

24. Apply antibiotic ointment (i.e., Vetropolucine) directly onto the mouse eyes from the tube and coat the eye and remove surgical tape.
25. Monitor mice approximately every 15 min until fully recovered from anesthesia (See AAV Intravitreal Injection anesthesia recovery above).
26. Mice should receive analgesic, such as 0.1 mg/kg buprenorphine hydrochloride, subcutaneously every 8–12 h for 24 h post optic nerve crush.

***Note:*** Alternatively, subcutaneously injection of 1 mg/kg Buprenorphine sustained release (SR) can be used. One dose of Buprenorphine can last for 46–48 hours in mice.

See Troubleshooting 2.

***Optional:*** Depending on your experimental purpose, you may choose to investigate the rate, timing, or fate of OPCs that proliferate after specific times after injury. If this is not necessary for your given experiment, please omit Part 4 and proceed to Part 5, below.

### Part 4: BrdU injection

**Timing: [0.5 h]**

27. Conduct BrdU birth dating by administering BrdU via i.p. injection at a concentration of 100 mg/kg.
  ***Note:*** You can use BrdU for two different readouts:
  a. If interested in OPC proliferation rate at specific time points after injury, inject only once and wait 3 h before starting tissue collection (Part 6, below).
  b. If interested in rate or progress of OPC differentiation post injury by BrdU co-immunostaining, inject BrdU once daily for 7 days from day 3 to day 10 post injury. Harvest tissue when you are ready to conclude your experiment. (See Figure 2A in Wang et. al. 2020).
    ***Optional:*** CTB injection protocol is used if you wish to visualize the regenerating RGC axons within the optic nerve during your analysis. This injection should be conducted 2 days prior to euthanizing the mouse to harvest the optic nerve. If not needed, please omit Part 5 and proceed to Part 6, below.

### Part 5: Label regenerated axons

**Timing: [0.5–1 day depending on the number of animals]**

***Note:*** CTB injection protocol is extremely similar to the AAV intravitreal injections above.

28. Attach micropipette to the prepared Hamilton syringe and tubing.

***Note:*** Keep the CTB solution on ice during the injection.

29. Apply 1 drop of Tropicamide to mouse eyes to dilate pupil
30. Apply 1 drop of Proparacaine to mouse eyes for local anesthesia
31. Hold mouse head in left hand, so that its skin spreads away from eye.
32. Using a Bulldog Serrefines clamp skin surrounding back of eye so that eye protrudes out (See AAV intravitreal injections, above for more detail)
33. Place mouse head under microscope at a 60º angle and stabilize the head with non-dominant hand.
34. Using your dominate hand, insert needle at a parallel angle to the eye.

***Note:*** You should be able to see the previous injection site from AAV administration. Try to insert the micropipette at the same location. The lens of a mouse eye is much larger than human or dog (see Figure 3**A**, and Intravitreal AAV Injection above).

35. Remove ∼1 μL of vitreous humor from eye using Hamilton syringe
36. This is done to reduce spilling and intraocular pressure from the upcoming injection.

***Note:*** Aseptic technique requires using one needle to remove liquid and another for injection

37. Pipette 1–2 μL of CTB into microinjection needle using Hamilton syringe
  a. Make sure there are no bubbles in the needle or syringe to ensure smooth and good injection.
38. Re-insert needle back into back of eye (via hole from 1^st^ insertion) and slowly inject CTB into vitreous space eye

***Note:*** Leave needle in place for a few seconds to allow CTB to diffuse and reduce loss when micropipette is removed.

39. Rotate the mouse and repeat for 2^nd^ eye.
40. Use cotton bud to remove any blood.
41. Apply antibiotic ointment (i.e., Vetropolucine) directly onto the mouse eyes from the tube.
42. Monitor mice approximately every 15 min until fully recovered from anesthesia.
43. Remain with animals until all mice awaken and can then return cages to housing unit.
44. Monitor mice daily for 7–10 days, and then twice a week for long term, per your ethical committee post-operative protocol.

### Part 6: Optic Nerve Dissection

**Timing: [0.5 h per mouse]**

45. Tissue can be harvested at variable time points after ONC, depending on read-out of interest and treatments protocol you are testing.

***Note:*** As a reference point, relatively robust myelination was observed ∼48 days post-crush with the treatments outlined in Wang *et al.* 2020.

**Figure 4:**
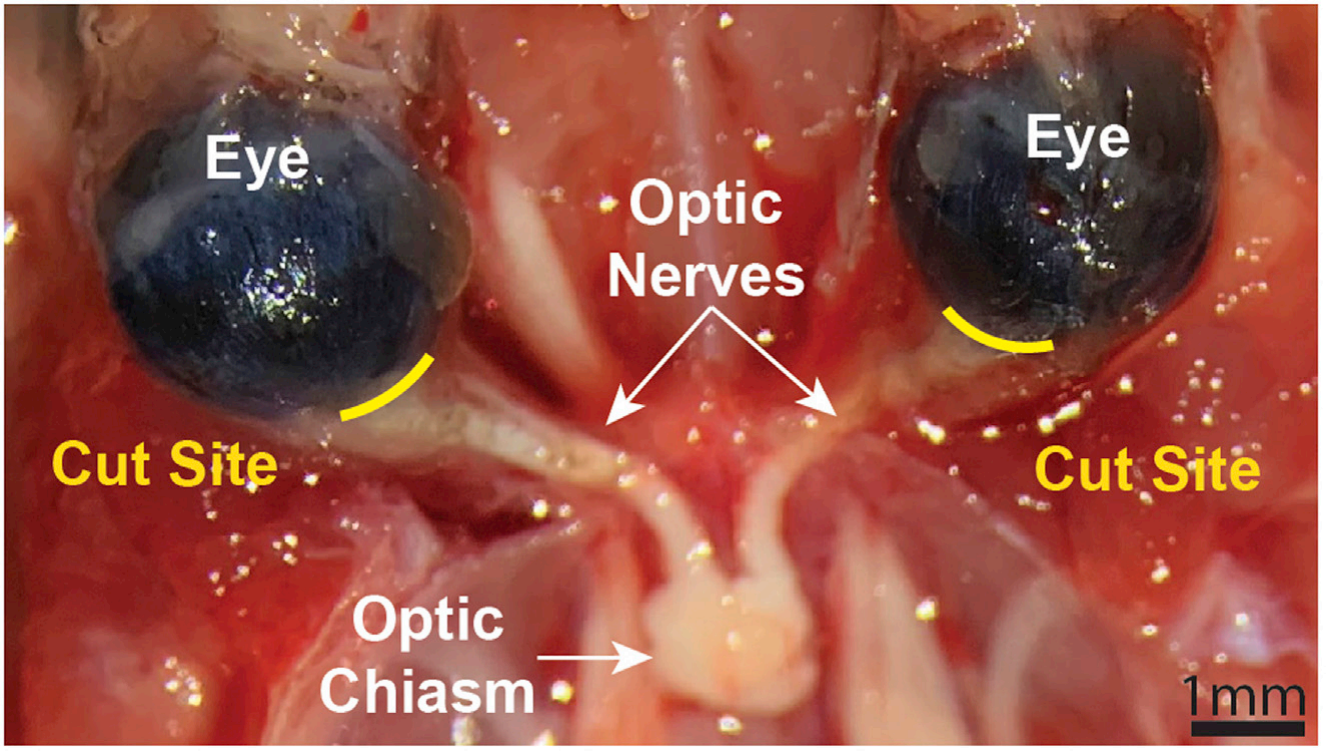
Optic nerve dissection View of the optic nerves once the brain and side skull bones have been removed (Step 48). Yellow lines indicate the cut sites to detach the nerve from eye. It is important to cut as close to the eye as possible to preserve the crush site in the nerve sample. Crush site will only be ~ 500 μm back from the eye. Scale Bar = 1 mm
  c. Move under a dissecting microscope
  d. Using forceps, carefully break and pull away the remaining bone around the eye socket.
    ***Note:*** The skull bone can accidentally damage the optic nerve if not removed properly. Make sure to pull the bone AWAY from the eye and nerve by pulling bone fragment in towards the empty skull cavity (where the brain was located) and not down or out towards the base of the skull or eye (respectively). This will help prevent accidental damage to the nerve.
  e. Using curved Student Vanna spring scissors (F.S.T. #91501-09), cut the eyelid way from the eye so its separated
  f. Looking through the scope, locate the ocular muscle that sits on top of the eye and cut
    i. This will cause the muscle to pull back and expose the optic nerve as it enters the eye.
  g. Cut the optic nerve as close to the back of the eye as possible using the spring scissors (Figure 4).
    **CRITICAL:** The crush site is only ∼500 μm - 1 mm back from the eye, so it is essential that you cut at the most distal part of the optic nerve right as it enters the eye so that you have the crush site in your analysis.
  h. Cut the optic chiasm in half so each optic nerve can be separated
  i. Using fine forceps, grasp the end of the nerve at the optic chiasm and gently pull.
    i. Should the optic nerve be properly cut, it should slide out through the muscle and eye socket without any resistance.
    ii. For picking up or handling of the optic nerve using forceps, be sure to grab the nerve near the optic chiasm, which is far enough from the crush site. This will prevent damaging the tissue around the crush site. The optic chiasm is not used for the later analysis.
  j. Store optic nerves in 1×PBS in 24 well plates at 4°C
    **Pause point:** Tissue can be stored under these conditions for ∼ 1 week until ready to proceed.

46. Give animals an i.p. of anesthesia (Ketamine-Xylazine Mixture).
  a. Use paw and tail pitches to confirm the animal is fully anesthetized

47. Perfuse the mouse:
  a. Pin the mouse down using needles through each paw so the animal is splayed on their back with their chest facing you.
  b. Incise the musculature immediately caudal to the sternum.
  c. Cut the ribs bilaterally and then completely cutting the diaphragm.
  d. Insert needle into left ventricle and pierce right atrium with sharp fine scissors.
  e. Perfuse the animal transcardially with ∼100 mL PBS until no blood is seen following out of the left ventricle.
  f. Perfuse with ∼100 mL 4% PFA
    ***Note:*** Flow rate of perfusion should be so that it takes ∼1–2 minutes to administer 100mL of the PFA.
  g. Separate head of the animal and place in Falcon tube containing 4% PFA.
  h. Passively fixate the head at 4°C for 8–12 h.
  i. Wash off PFA the following morning using 1×PBS for 3**×**10 min
    ***Note:*** Over fixation of the tissue can lead to later issues with immunohistochemical staining, such as increased background noise, or loss of signal.

48. Dissect out the optic nerves from the head:
  a. Remove skin/fur from the skull.
  b. Insert the tip of small scissors into the exposed back of the brainstem (starkly white tissue).
    i. Keeping scissors angled upward, cut skull in a straight line through the bregma to the front of the brain.
    ii. Using forceps peel and break off the skull to expose the brain.
    iii. With the forceps, grab the brainstem / cervical spinal cord region, and gently lift and flip the brain up and out of the skull cavity.
    iv. Optic nerves (connected to the optic chiasm) should be seen resting at the base of the skull (Figure 4).

### Part 7: Immunohistochemistry & analysis

**Timing: [5–6 days]**

**Figure 5:**
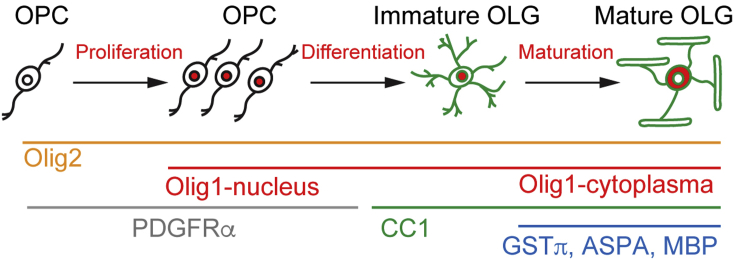
Oligodendrocyte maturation and markers Schematic depicts the major steps of maturation that occur from OPCs to mature, myelinating oligodendrocytes. It also highlights some markers that can be used to identify oligodendrocytes at different stages of this maturation.

***Note:*** Please see Figure 5 for the expression pattern of the Oligodendrocyte lineage.

49. Place the dissected optic nerves into 30% sucrose (with 1×PBS) for ∼48 h at 4°C to cryoprotect the tissue.

***Note:*** Tissue will sink to the bottom of the well when it is ready to proceed to the following steps.

50. Remove excess sucrose from the surface of the optic nerves using a Kimwipe to absorb the liquid prior to embedding.
51. Embed the optic nerves in Optimal cutting temperature compound (OCT) for cryo-sectioning. Make sure the nerve is laid flat so that the nerve can be sectioned evenly along the longitudinal direction.
52. Section optic nerves at 10 μm thickness and attach sections on to pre-coated glass coverslips for immunohistochemistry.

**Pause point:** Slides can be stored at −80°C until ready for staining. Short term (<1 month) storage at −20°C is also acceptable.

53. Bring slides to room temperature
54. Wash slides in 1×PBS to remove OCT from the slide.

See Troubleshooting 3.

***Optional:*** If not staining for BrdU, step 55 can be omitted.

55. If staining for BrdU, denature tissue sections.
  a. Place tissue in HCl (2 N) for 30 min at 37°C.
  b. Then neutralize with 0.1 M sodium borate buffer for 10 min
  c. Then proceed to the normal staining procedure
56. Place tissue sections in blocking buffer for 1 h at room temperature.
  a. 0.5% Triton X-100 and 10% normal donkey serum in PBS
57. Replace solution with primary antibody solution in blocking buffer and incubate at 4°C in a humidifying chamber for 8–12 h.

See Troubleshooting 4.

58. Remove primary solution and wash slides in 1×PBS
  a. Repeat for a total of three 10 min washes
59. Add secondary antibodies in blocking buffer for 2 h at room temperature in a humidifying chamber.
  a. Choose secondaries that correspond to the primary antibodies used.

***Note:*** Be sure to avoid secondaries in the red channel (555-, 594-, or TexasRed) as you will be unable to discern the tamoxifen induced tdTomato+ oligodendrocyte lineage cells from your other antibody marker(s).

60. Wash off secondary antibody solution in 0.1% Triton X-100 in 1×PBS.
  a. Repeat for a total of three 10 min washes
61. Dry slide as much as possible without allowing the tissue to dry out
62. Mount slides with DAPI-containing mounting solution and sealed with glass coverslips and clear nail polish.
63. Allow slides to dry completely before imaging.

**Pause point:** Slides can be stored at 4°C until ready for imaging.

**Figure 6:**
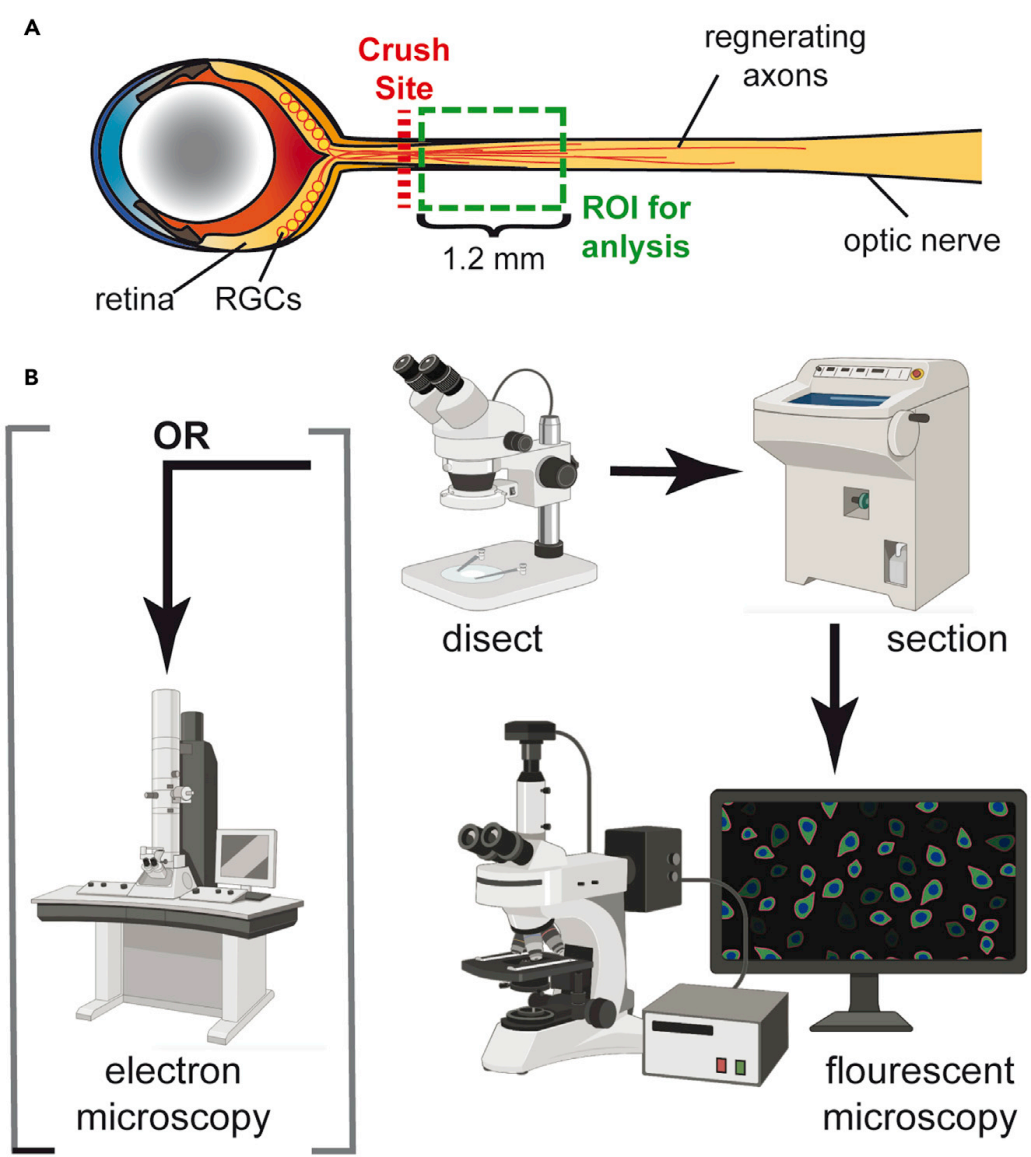
Analysis of optic nerves (A) Schematic shows the anatomy of the eye, retina, and optic nerve. Retinal ganglion cells (RGCs) with their regenerating axons are shown. Red dashed line indicates the crush site. Green box indicates the region of the optic nerve that should be used for analysis. (B) Flow chart depicting major steps to analyze optic nerve at the conclusion of the experiment.
  d. For each biological sample, 3–5 sections per optic nerve should be imaged for analysis.
65. Quantify your images:
  a. Quantifying Positive cells
    i. Quantify positive cells manually using the Plugin Analyze / Cell Counter
  b. For fluorescent intensity analysis:
    i. Convert images to 8-bit depth in ImageJ software.
    ii. Calculate mean intensity value using the built-in function: Analyze/Measure.

***Note:*** Alternatively, tissue can be used for *in situ* hybridization, iDISCO optical clearing (Renier *et al.*, 2014), or electron microscopy (Figure 6**B**). Choice of processing and analysis type will depend on the readout of interest for a given experiment.

64. Image sections for quantification:
  a. Use a confocal microscope for best cellular resolution
  b. Use a 10× or 20× objective.
  c. Image within 1.2 mm distal to the crush site because this area will have the highest proportion of regenerating axons and it is important to maintain a consistent location of analysis (see Figure 6A).

## Expected outcomes

This protocol will generate immunohistological data that will allow for the quantification of oligodendrocyte proliferation, differentiation, and myelination after an optic nerve injury. This protocol generates a simplified and reproducible *in vivo* CNS injury system that allows for easy testing and analysis of remyelination treatments. Due to the complete lack of myelination on the regenerating axons after ONC, any observed myelination can be attributed to the treatments being tested. This protocol will generate quantitative data on the effectiveness of myelination treatments before moving into testing in more complex model systems such as EAE, Leukodystrophies, or spinal cord injury.

## Quantification and statistical analysis

To analyze images collected from this protocol please do the following when running statistical analysis using software Prism-GraphPad: Two-tailed Student’s t test should be used for single comparisons between two groups. Other data should be analyzed using one-way or two-way ANOVA depending on the appropriate design. Post-hoc comparisons are carried out only when the primary measure showed statistical significance. P values of multiple comparisons should be adjusted using Bonferroni’s correction. Represent error bars as mean ± SEM in all figures. Mice with different litters, body weights, and sexes should be randomized and assigned to different treatment groups. No other specific randomization should be used for the animal studies.

## Limitations

Post injury, there is myelin debris accumulation and pre-existing oligodendrocytes within the optic nerves. It is critical to distinguish between those remaining oligodendrocytes versus the newly generated oligodendrocytes and myelination. Commonly used single marker staining would not be able to solve this problem. While BrdU/CC1 double staining in wild type mice could map some new oligodendrocytes post injury, using an OPC lineage tracing mouse line is more definitive. Reporter lines such as PDGFRα^CreER^;tdTomato mice, are more reliable for visualizing new oligodendrocytes and new myelin post optic nerve injury. However, these Cre lines may have certain leakiness and should be tested after crossing with specific reporters.

While axon regeneration is needed for testing the pro-myelination effects, injured optic nerves per se could be valuable for studying other events such as myelin debris clearance and microglia phenotypic changes.

## Troubleshooting

### Problem 1

Our unpublished results indicated that the PDGFRα^CreER^ line (Kang et al., 2010) can have leaky expression, in the absence of tamoxifen induction, when crossed to sensitive reporter lines such as Ai14 (Madisen et al., 2009), or Tau-GFP (Hippenmeyer et al., 2005) (step 1 of Part 0 in before you begin).

### Potential solution

We recommend using the RTM tdTomato line (Arenkiel et al., 2011) when working with PDGFRα^CreER^. If you are interested in total myelin present in the nerve, regardless of the timing of its generation, you can use the PLP^CreER^ line (JAX #: 005975) in conjunction with the Ai14 tdTomato line (JAX #: 007914) or Tau-GFP (JAX #: 021162) reporter lines (Doerflinger, Macklin and Popko, 2003; Hippenmeyer et al., 2005; Madisen et al., 2009). But be conscious of the difference in oligodendrocyte populations that you are labeling between the PLP^CreER^ and PDGFRα^CreER^ lines.

### Problem 2

Mouse eye appears cloudy/opaque in the days following the ONC (End of Part 3, Optic Nerve Crush).

### Potential solution

Cloudy eyes indicate that the crush conducted on the eye was too severe. Do not use an optic nerve from a cloudy eye for your analysis. This can increase the amount of variance in your analysis and therefore should be avoided. Try to ensure to use the same consistent pressure with every crush, and plan you experiment accordingly with a few extra animals to account for this possibility. If this is a recurring issue, make sure you reduce the pressure of the crush. You can also choose to use different self-closing Fine Forceps (F.S.T. Cat #: 11487-11) which will provide consistent pressure. If using the self-closing fine -forceps, be sure to replace with a fresh pair every ∼6 months as the closure force will weaken over time.

### Problem 3

Sectioned optic nerve tissue can often have difficulty remaining stuck to slides (even positively charged slides) (step 54 of Part 7).

### Potential solution

We recommend using Fisher Brand Superfrost Plus Gold, Catalog #: 15-188-48 for optic nerve sections. However, if sections are still coming loose during the staining procedure, we recommend placing slide on a warm plate set at 37°C for 2 h before first PBS wash to remove the OCT (Step 54).

### Problem 4

The endogenous tdTomato signal from the Arenkiel et al., line is weak and bleaches easily when imaging (Step 57 of Part 7).

### Potential solution

We recommend using an RFP primary antibody and a 594 secondary to amplify and stabilize the tdTomato reporter signal (See key resources table for RFP antibody information) (Step 57).

## Resource availability

### Lead contact

Further information and requests for resources and reagents should be directed to and will be fulfilled by the lead contact, Dr. Zhigang He: (zhigang.he@childrens.harvard.edu).

### Materials availability

Please contact Dr. Zhigang He (zhigang.he@childrens.harvard.edu) to inquire about access to other materials in this manuscript.

## Data Availability

This study did not generate any new code. The data sets supporting the current study have not been deposited in a public repository because they are included in the original publications, Wang et al., 2020 and Bei et al., 2016 but are available from the corresponding author upon request.
